# The Elements of Physiological Physics; an Outline of the Elementary Facts, Principles, and Methods of Physics; and Their Applications in Physiology

**Published:** 1884-12

**Authors:** 


### Notes on Books.

*The Elements of Physiological Physics; an outline of the Elementary Facts, Principles, and Methods of Physics; and their applications in Physiology.* By J. M'Gregor-Robertson, M.A., M.B., C.M. London: Cassell and Company. 1884.

This volume, the latest of the "Manuals for Students of Medicine" published by Messrs. Cassell and Company, forms a companion volume to others of the same series. The first of these, Klein's *Elements of Histology*, has already become well known amongst students, and has achieved a considerable popularity amongst the class for whom it was intended. The second of them, Power's *Elements of Human Physiology*, gives in less than four hundred pages a general outline of the physiology of man, the details of structure being passed over in silence, and all descriptions of instruments and methods of procedure being omitted. The third, on *Physiological Physics*, comprises 528 pages, and has 219 wood engravings. This volume supplements the omissions of the others, giving descriptions of instruments and details as to their use. The author has also given the most important elementary facts and principles of Physics as a preliminary to the more detailed descriptions of the physical apparatus and methods adapted to physiological purposes. To the average medical student, to those who aspire to the higher degrees, and more especially to those who intend to become familiar with the modern methods of investigation of physiological and pathological problems, this book will be an invaluable companion. It will to some extent compensate for the want of familiarity with the subject of Experimental Physics as it is now taught in many of our "University Colleges and Technical Schools." A course of Modern Physiology should, if the time of the student permitted, commence with such a course of Physics and Chemistry as will enable him to understand, and utilise for purposes of research, the various physical and chemical methods on which the modern development of Physiology has depended. The want of this knowledge is a constant source of annoyance both to the student and the teacher, and accordingly both the study and the teaching of Physiology have become increasingly difficult because of the broadening of the basis on which the science is now built. It has been frequently remarked that a mere sight of the pages of a modern journal of Physiology is sufficient for the average practitioner, who will scarcely pretend to understand, or attempt seriously to read, pages which would be almost as intelligible if written in an unknown language.

In this book the various branches of Physics are dealt with in order—electricity, magnetism, hydrostatics, hydrodynamics, pneumatics, optics, acoustics, heat, and dynamics. The experiments described are those commonly employed in the elucidation of physiological problems, and they are described in such a way that the book may be used as a hand-book for the laboratory, by means of which the student may work out the experiments for himself. The size of the volume is an indication of the increasing importance of accurate methods in physiological work, and it contrasts remarkably with that by Mr. Power, which includes the whole range of Physiology in about two-thirds the number of similar pages.

The series of red-cover and red-edge volumes which comprise the field commonly included in Physiological Text-books will not be complete without the work by Dr. Ralfe on *Clinical Chemistry*, and that by Professor Jeffrey Bell on *Comparative Physiology and Anatomy*. Armed with this series of monographs, the student should be able to master all the essential points in the subject of Physiology; but it may be questioned whether they will enable him to dispense with the beautifully illustrated eleventh edition of Kirke's *Hand-book* or with the still more enchanting Foster's *Text-book*.

